# The effectiveness of mental health courts in reducing recidivism and police contact: a systematic review protocol

**DOI:** 10.1186/s13643-016-0291-8

**Published:** 2016-07-27

**Authors:** Desmond Loong, Sarah Bonato, Carolyn S. Dewa

**Affiliations:** 1 Centre for Research on Employment and Workplace Health, Centre for Addiction and Mental Health, 33 Russell Street, Toronto, M5S 2S1 Canada; 2 Library Services, Centre for Addiction and Mental Health, 33 Russell Street, Toronto, M5S 2S1 Canada; 3 Department of Psychiatry and Behavioral Sciences, University of California, Davis, 2103 Stockton Blvd, Sacramento, CA 95817 USA

**Keywords:** Mental health courts, Recidivism, Rearrests, Police contact

## Abstract

**Background:**

Mental health courts were created to help criminal defendants who have a mental illness that significantly contributes to their criminal offense. Despite the increasing number of mental health courts around the world, data about their effectiveness have only begun to emerge in the past decade. The purpose of this systematic literature review is to assess the current evidence on the effectiveness of mental health courts. Specifically, this review will address the question, “How effective are mental health courts in reducing recidivism and police contact?”

**Methods/design:**

Eight electronic databases will be searched, specifically PsycINFO, Medline, Medline In-Process, Embase, Web of Science, CINAHL, Social Work Abstracts, and Criminal Justice Abstracts. A multi-phase screening process will be used to identify relevant search hits. Articles that pass the three-stage screening process will then be assessed for risk of bias and have their reference lists hand searched. Full-text articles that are rated to have low to moderate risk of bias will be summarized into two tables, one containing a brief description of the study and the other reporting the results of relevant outcomes measured.

**Discussion:**

By synthesizing the results of the studies, this systematic review will help illuminate gaps in the literature, direct future research, and inform policy makers.

**Systematic review registration:**

PROSPERO CRD42016036084

## Background

In the mid-1990s, courts across Canada and the USA reported significant increases in the number of defendants with mental illness entering the criminal court system [1, 2]. In some jurisdictions, this increase has been in excess of 10 % per year [2]. As a response to this growing problem, mental health courts were created to help criminal defendants who do not meet not-guilty-for-reason-of-insanity criteria but who have a mental illness that is a significant contributing factor to their criminal conduct [3].

Mental health court diversion programs are characterized by three key components: screening, assessment, and negotiation between court diversion and criminal justice staff [4]. Screening involves the identification of defendants who are suspected of having a mental illness. Assessment involves the evaluation of identified defendants by a mental health professional. The last component involves court diversion staff negotiating with prosecutors, defense attorneys, the courts, and community-based mental health providers to work towards having charges reduced or even waived [4].

Although the number of mental health courts continues to increase across North America and abroad, data have only begun to emerge in the past decade suggesting that mental health courts reduce recidivism and improve client outcomes [1, 2].

## Purpose of systematic review

The purpose of this systematic review of the literature is to look at the current evidence on the effectiveness of mental health courts in reducing client rearrest rates and contact with police. Specifically, this review will address the question, “How effective are mental health courts in reducing recidivism and police contact?” This review will contribute to the literature by examining the evidence on whether mental health court clients benefit from their linkage to mental health services in the community by helping them live in the community independently and stay out of the legal system.

## Previous reviews and rationale

Although systematic reviews on mental health courts have been published, with 2011 and 2015 being the most recent [5–7], there are several limitations with these three previous reviews that this systematic review will address.

Firstly, this systematic review will report on the current evidence on the effectiveness of mental health courts by collecting data on peer-reviewed studies up until February 2016. The 2011 reviews by Sartechi et al. [6] and Lange et al. [5] are based on data collected up until July 2009 and January 2011, respectively. The 2015 paper by Honegger [7] reviews data collected up to August 2014.

Secondly, this review will employ a more comprehensive search strategy than previous reviews. This review will use truncated word search commands and database-specific adjacency operators that were not used by previous reviews. Truncated search commands broaden a search strategy to capture variations in spelling for a particular word. This means a keyword can be comprehensively searched in one single command as opposed to the multiple search commands required to account for each variation of spelling. Adjacency commands, on the other hand, help to look for a string of words that are within a specified number of words apart. This allows key phrases to be searched without having to worry about the order of words the author(s) may use and all the different combinations.

Lastly, this review will not be using geographical search limitations. Previous reviews focused on specific geographical locations, such as North America [5] or the USA [7]. Given mental health courts exist in many jurisdictions around the world [1], the use of geographical limitations are arguably too narrow of a view.

## Methods/design

This systematic literature review will be reported following the Preferred Reporting Items for Systematic Reviews and Meta-Analyses (PRISMA) guidelines [8]. Because this review will only use publically available information, an ethics review board approval will not be required.

## Eligibility criteria

For the purposes of this review, mental health courts will be defined as specialized courts dedicated to persons with serious mental illness who have committed a crime [2]. Court support services will be defined as services provided to clients to help navigate the court system and utilize mental health services [9]. Recidivism will be defined as rearrests, and police contact will be defined as any kind of client involvement with police in the community for suspected violations of the law by the client (as opposed to contacts resulting from being a victim of a crime).

The following eligibility criteria will be used to screen for relevant peer-reviewed articles:

1. The study reports on a mental health court(s).
2. The study reports on adults (18 years or older) with mental disorders who have been charged for committing a crime.
3. The study reports program outcome measures on recidivism and/or police contact.

The following exclusion criteria will be used:

1. The study reports only on juvenile courts.
2. The study reports solely on drug courts.
3. The study population does not have identified mental disorders.
4. There are no outcome measures reported.
5. There is no comparison group.
6. The article is not reporting on original research.
7. The study is a case study.
8. The study only re-reports findings from an already included publication of the same author using the same dataset.

## Search strategy

### Electronic databases

In total, eight electronic databases will be searched for this systematic review:

1. PsycINFO (an index of journal articles, books, chapters, and dissertations in psychology, social sciences, behavioral sciences, and health sciences)
2. Medline (an index of biomedical research and clinical sciences journal articles)
3. Medline In-Process (an index of biomedical research and clinical sciences journal articles awaiting to be indexed into Medline)
4. Embase (an index of biomedical research and abstracts from biomedical, drug, and medical device conferences)
5. Web of Science (an index of journal articles, editorially selected books, and conference proceedings in life sciences and biomedical research)
6. CINAHL (an index of journal articles, books, dissertations, and conference proceedings in nursing, biomedicine, health sciences librarianship, alternative medicine, consumer health, and allied health disciplines)
7. Social Work Abstracts (an index of abstracts in social work and human services)
8. Criminal Justice Abstracts (an index of abstracts in criminal justice and criminology)

Specific to each database, the finalized search strategies will be developed and executed with a professional health science librarian. Medline, Medline In-Process, PsycINFO, Embase, and Social Work Abstracts will be searched using the OVID platform. Web of Science will be searched using the Thomson Reuters search interface. Lastly, CINAHL and Criminal Justice Abstracts will be searched using the EBSCO platform. Across all databases, search results will be limited to English language journals and published articles in peer-reviewed journals whenever possible. Search results will not be limited by publication year.

### Search term development

PsycINFO will be used to develop the finalized search terms for this systematic review. The main rationale is because PsycINFO has the largest index of journals relating to both mental health and justice. In the first step, a search will be executed using the preliminary keywords shown in Table 1. Keyword searches are particularly useful when the subject heading for a particular topic is not known and allows for queries of keywords that appear anywhere in an article’s index record [10]. The results of the preliminary keyword search will then be screened for relevancy using the eligibility criteria outlined previously. Articles that are found to be relevant will be examined for the keywords under which they are indexed. PsycINFO subject headings will also be identified to broaden the search if required. The development of search terms will stop when no new keywords are found from newly identified articles. Each new article, identified with each new tested term, will be examined for new relevant search terms. In an iterative process, every keyword will be tested individually for inclusion into the search strategy. In the last step, the final search strategy will be adapted and executed for the remaining seven databases. When searching in Medline, Medical Subject Headings (MeSH) will be examined. MeSH terms are important to consider because they are the US National Library of Medicine’s predefined and authorized vocabulary thesaurus used to index journal articles in Medline and PubMed [11]. Because MeSH terms follow a hierarchical structure, these terms allow for searches at various levels of specificity [11]. That is, all terms follow a tree structure from broad to specific, and this will allow for broadening the search if needed. Preliminary search strategies for each of the databases are included in Tables 1, 2, and 3.

**Table 1:** OVID preliminary search strategy (Medline, Medline In-Process, PsycINFO, Embase, and Social Work Abstracts)

	Search terms
1.	(mental$ adj3 health$ adj3 court$).mp.
2.	(mental$ adj3 health$ adj3 justice$).mp.
3.	(mental$ adj3 ill$ adj3 court$).mp.
4.	(mental$ adj3 ill$ adj3 justice$).mp.
5.	(court$ adj3 diversion$).mp.
6.	(jail$ adj3 diversion$).mp.
7.	(post$ adj3 booking$ adj3 diversion$).mp.
8.	1 OR 2 OR 3 OR 4 OR 5 OR 6 OR 7

*Note*: $= Database specific truncation search command

**Table 2:** Thomson Reuters preliminary search strategy (Web of Science)

	Search terms
1.	mental* NEAR/3 health* NEAR/3 court*
2.	mental* NEAR/3 health* NEAR/3 justice*
3.	mental* NEAR/3 ill* NEAR/3 court*
4.	mental* NEAR/3 ill* NEAR/3 justice*
5.	court* NEAR/3 diversion*
6.	jail* NEAR/3 diversion*
7.	post* NEAR/3 booking* NEAR/3 diversion*
8.	1 OR 2 OR 3 OR 4 OR 5 OR 6 OR 7

*Note*: *= Database specific truncation search command

**Table 3:** EBSCO preliminary search strategy (CINAHL and Criminal Justice Abstracts)

	Search terms
1.	mental* N3 health* N3 court*
2.	mental* N3 health* N3 justice*
3.	mental* N3 ill* N3 court*
4.	mental* N3 ill* N3 justice*
5.	court* N3 diversion*
6.	jail* N3 diversion*
7.	post* N3 booking* N3 diversion*
8.	1 OR 2 OR 3 OR 4 OR 5 OR 6 OR 7

*Note*: *= Database specific truncation search command

### Study selection

A multi-phase screening process will be used to identify relevant search hits using the eligibility criteria mentioned previously. Phase 1 will involve screening articles by title. Citations that pass the first phase will then be evaluated for relevance based on their abstracts. The full-text articles that pass the first and second screening will be evaluated for content. The entire multi-phase screening process will be done independently by two reviewers. Using Cohen’s kappa coefficient (ƙ), the inter-rater reliability between both raters will be calculated and corrected for chance [12].

Articles with rating disagreements will be discussed until a consensus is reached. The reference lists of all accepted studies will also be hand searched. Articles identified through this process will be subjected to the same multi-phase screening process described previously using the same eligibility criteria.

### Risk of bias assessment

Articles that pass the three-stage screening process will then be assessed for risk of bias. A 6-item risk of bias checklist adapted from Cochrane [13] and Dewa et al.’s [14] Risk of Bias Assessment tool will be used:

1. Adequate sequence generation
  Group assignments of participants follow rules that are based on chance.
2. Allocation concealment
  Schedule of random assignments are kept concealed from personnel involved in study enrollment.
3. Blinding
  Participants and personnel are masked of the knowledge of which intervention was received.
4. Incomplete outcome data
  There is no significant difference between groups who withdraw from the study.
5. Selective reporting
  Study results are not selectively reported.
6. Recruitment strategy
  The recruitment process is open to all potential participants who meet the study eligibility criteria.

Each of the six aforementioned criteria will be given one of three possible scores: −1 (if there is a high risk of bias), +1 (if there is a low risk of bias), or 0 (if there is not enough information to assess risk). The minimum and maximum for any one article is −6 and +6, respectively. Total scores of 2 and below will be categorized as high risk of bias and scores between 3 and 4 points will be considered as moderate risk. Articles that score 5 points and above will be considered as low risk of bias.

### Synthesis

All full-text articles that are rated as low to moderate risk of bias will be summarized in two summary tables. The first summary table will contain a brief description of the study. This will include the name of the authors, the journal name, the year the study was published in, description of the study population, the study design employed, and the type of recidivism or police outcomes that were measured. The second summary table will report the results of the outcomes that were measured.

## Discussion

This systematic literature review seeks to examine the current evidence on the effectiveness of mental health courts. Specifically, it seeks to answer, “How effective are mental health courts in reducing recidivism and police contact?” This review will illuminate gaps in the evidence with respect to recidivism and police contacts and also help guide the direction of future research. Furthermore, this review will help inform policy makers in the establishment or continuation of mental health courts in their respective jurisdictions.

## Abbreviations

PRISMA, Preferred Reporting Items for Systematic Reviews and Meta-Analyses
